# Methodological Issues in Randomized Clinical Trials for Prodromal Alzheimer's and Parkinson's Disease

**DOI:** 10.3389/fneur.2021.694329

**Published:** 2021-08-06

**Authors:** Camila Henriques de Aquino

**Affiliations:** ^1^Hotchkiss Brain Institute, University of Calgary, Calgary, AB, Canada; ^2^Department of Clinical Neurosciences, University of Calgary, Calgary, AB, Canada; ^3^Department of Health, Evidence and Impact, McMaster University, Hamilton, ON, Canada

**Keywords:** clinical trials, prodromal, Alzheimer's disease, Parkinson's disease, trial design, methodology, prodromal AD, prodromal PD

## Abstract

Alzheimer's disease (AD) and Parkinson's disease (PD) are the first and second most common neurodegenerative disorders, respectively. Both are proteinopathies with inexorable courses and no approved disease-modifying therapies. A substantial effort has been made to identify interventions that could slow down the progression of AD and PD; to date, with no success. The advances in biomarker research improved the identification of individuals at risk for these disorders before symptom onset, recognizing the pre-clinical stage, in which there is abnormal protein accumulation but no clinical symptoms of the disease, and the prodromal stage, in which mild symptoms are present but the clinical diagnostic criteria for disease cannot be fulfilled. The ability to detect pre-clinical and prodromal stages of these diseases has encouraged clinical trials for disease-modification at earlier phases, seeking to slow or prevent phenoconversion into clinical disease. Clinical trials at these stages have several challenges, such as the identification of the eligible population, the appropriate choice of biomarkers, the definition of clinical endpoints, the duration of follow-up, and the statistical analysis. This article aims to discuss some of the methodological challenges in the design of trials for pre-clinical and prodromal phases of AD and PD, to critically review the recent studies, and to discuss methodological approaches to mitigate these challenges in trial design.

## Introduction

Alzheimer's disease (AD) and Parkinson's disease (PD) are the first and second most common neurodegenerative disorders, respectively. It is estimated that nearly 45 million people live with AD and related forms of dementia globally (1, 2), and that other 6.1 million live with PD (2); both with prospects for an exponential increase in prevalence due to population growth and prolonged lifespan (3, 4). While these are distinct disorders in terms of pathology, clinical presentation, and management, there are essential points in common: both are proteinopathies with an inexorable course and no definite disease-modifying therapy (DMT) (5, 6).

DMT is defined as an intervention that will delay, slow down, or halt the progression of a disease (7). In the field of neurodegenerative disorders, a substantial effort has been made over the past decades to identify an intervention that could target the key mechanisms that ultimately lead to cell death; to date, with no success (8, 9). This is an intriguing situation, notably when experimental studies have unveiled so many promising drugs, which ultimately failed in human phase 2 or phase 3 clinical trials.

Several reasons have been speculated for this challenging translation of laboratory results into clinical benefit, including but not limited to the bioavailability of the compounds within the central nervous system (CNS), the target engagement, the poor tolerability to the interventions, and importantly, the design of the randomized controlled trials (RCTs) (10).

The timing of intervention has been a significant source of criticisms once the vast majority of the RCTs in AD and PD have enrolled participants with established clinical diagnoses, i.e., when symptoms were fully developed. It is well-known that in the clinical stages, even when symptoms are mild, the neurodegenerative process is already advanced and most likely irreversible (8, 9). The currently proposed strategy is to address the efficacy of putative DMT in the pre-clinical and prodromal phases of AD and PD, which means, before the onset of clinical symptoms. However, several methodological challenges may arise in the design of such trials, including the identification of the eligible population, the appropriate choice of biomarkers for subject enrollment, the definition of clinical endpoints, the potential need for surrogate outcomes, the definition of patient-reported outcomes, the duration of follow-up, and the statistical analysis (9).

Considering these challenges, this article aimed to discuss the methodological challenges in the design of RCTs for pre-clinical and prodromal phases of AD and PD, respectively; to critically review the recent RCTs performed for DMT; and to discuss potential methodological approaches to mitigate the issues in trial design.

## Overview of the Clinical Conditions

### Alzheimer's Disease

AD is a neurodegenerative disorder responsible for approximately 60% of all dementia cases worldwide (11), with a high prevalence of 10 to 30% in the population older than 65 (6). The pathology is marked by the accumulation of insoluble and proteolysis-resistant forms of amyloid-beta (Aβ) forming plaques in the extracellular spaces and blood vessel walls, combined with aggregation of hyperphosphorylated tau protein within the neurons forming neurofibrillary tangles (12). These abnormalities lead to impaired synaptic plasticity, synaptic loss, decrease in specific neurotransmitters, and ultimately to selective neuronal death (6, 13). The Aβ peptide fibrils are produced by the sequential cleavage of the amyloid precursor protein (APP) by the enzymes β-secretase (BACE-1) and γ-secretase, the latter encoded by the presenilin 1 (*PSEN1*) and presenilin 2 (*PSEN2*) genes (14).

The clinical spectrum of AD can be heterogeneous, and two main clinical syndromes are recognized: Amnestic AD, with significant impairment of learning and recall, i.e., memory, and non-amnestic AD, which presents with impairment of language, visuospatial, or executive function (15). For the dementia diagnosis, a decline from the previous cognitive level, affecting at least two domains, and impacting the ability to function at work or usual activities is required (15). However, it has been recognized that the AD pathological process starts ~20 years before the development of dementia (16).

The current AD model recognizes three stages of the disease. The *pre-clinical stage* is defined by amyloid accumulation but intact cognitive abilities. The *prodromal stage* is defined by amyloid accumulation and the development of mild cognitive impairment (MCI); however, with no impact on the ability to function, which marks the transition to the dementia phase (6, 17). A study estimated the duration of the pre-clinical phase in ~10 years, and the prodromal phase in 4–5 years, which corresponds to a window of opportunity to intervene in the biological process of the disease (17).

### Parkinson's Disease

PD has an estimated prevalence of approximately 1% in people older than 65 years (3). The pathology is marked by the presence of the intraneuronal Lewy bodies which is formed by aggregates of misfolded alpha-synuclein (α-syn) (18–20). The α-syn spreading mechanism is unclear, but the leading hypothesis is that the abnormal α-syn behaves like a prion, propagating from cell-to-cell (21–23).

Clinically, patients with PD develop bradykinesia, tremor, muscle rigidity, gait, and balance impairment (24). Also, several non-motor symptoms, including cognitive impairment, neuropsychiatric symptoms, sleep disorders, and autonomic dysfunction, add to the disease's burden (25). As in AD, PD has a progressive course leading to disability and dependence on caregivers.

Similarly to AD, PD pathology starts two decades before disease presentation. The Braak model of disease progression suggests that α-syn aggregates can be initially detected in the olfactory bulb, dorsal motor nucleus of the vagus nerve, and in the enteric plexus (Stage 1) (26, 27). Subsequently, it ascends through the brainstem to involve the pons and the midbrain, which causes the degeneration of the dopaminergic neurons in the substantia nigra and the first symptoms of the disease (Stages 2 and 3) (26). On a later stage, the limbic system (Stage 4), the prefrontal cortex, and the primary motor and sensory cortex (Stages 5 and 6) are affected (26, 27). The Movement Disorder Society (MDS) recognizes the early forms of PD as *pre-clinical PD*, which is defined by the presence of neurodegenerative synucleinopathy without clinical symptoms, and *prodromal PD*, which is defined by the presence of early symptoms and signs before the classical PD can be diagnosed (28). Once again, these very early phases of the pathological process correspond to the ideal timing for intervention, intending to stop the spread of the disease throughout the CNS.

## History of Clinical Trials for Disease-Modification in AD and PD

Both AD and PD have complex pathophysiological mechanisms, still not completely understood. Certainly, one of the main challenges has been to correctly identify the treatment targets and appropriate dosages for the interventions.

In AD, based on the Aβ deposition hypothesis, several clinical trials aimed to reduce the amyloid levels either by inhibiting its production using BACE1 or γ-secretase inhibitors or by using monoclonal antibodies that bind to soluble forms of amyloid (Table 1). In general, all of these studies have failed to demonstrate clinical efficacy (29–31, 35, 38–43), and in some, there was a deterioration of cognition in the intervention group (29, 38–40). These findings suggest that either Aβ is a marker of the disease but not its cause or that lowering Aβ by inhibiting the action of the secretases might affect the normal function of these enzymes required by other brain processes (44). Interestingly, by the time of the review of this article, a drug called Aducanumab, an anti-amyloid monoclonal antibody received approval by the FDA, based on the reduction of amyloid deposition, despite of uncertain clinical effects in the ENGAGE and EMERGE clinical trials (45).

**Table 1:** Recent clinical trials for disease-modification in AD.

**Drug name**	**Mechanism of action**	**Population (disease stage)**	**Dose and administration route**	**Sample size**	**Clinical outcomes**	**Biomarkers measured**	**Results**	**Comments**
Aducanumab ENGAGE (NCT02477800) EMERGE (NCT02484547)	Monoclonal antibody that binds soluble forms of amyloid	Mild AD	3, 6 mg/kg, or placebo; Monthly IV	ENGAGE: 1,647 EMERGE: 1,638	Primary: CDR-SB score Secondary: MMSE, ADAS-Cog 13, ADCS-ADL-MCI	Amyloid PET	Terminated for futility	~35% of participants developed ARIA-E
Atabecestat (JNJ-54861911) (29)	BACE-1 inhibitor	Pre-clinical	5, 25 mg, or placebo; oral	557	PACC, RBANS		Greater cognitive deterioration in the intervention groups in comparison to placebo	Hepatic toxicity
Avagacestat (30)	γ-secretase inhibitor	Prodromal	50 mg escalating to 25 mg, or placebo; oral	263	ADAS-cog, ADCS-ADL, CDR-SB, MMSE, free and cued selective reminding test	Total tau, phosphorylated tau, Aβ_1−42_ in CSF; Brain MRI; Amyloid PET in a subset of participants	Terminated early. Minimal reductions in CSF amyloid and no significant treatment differences in the avagacestat arm vs. placebo	Adverse events: gastrointestinal tract adverse events, increases in non-melanoma skin cancer and reversible renal tubule effects
Bapineuzumab (31)	Monoclonal antibody directed at amyloid plaques and oligomers	Mild to moderate	0.5, 1.0, 2.0 mg/kg, or placebo; IV every 13 weeks	Study 1: 1,121 *APOE ε4* allele carriers Study 2: 1,331 non-carriers	ADAS-cog; DAD	Amyloid PET, phosphorylated tau in CSF	No efficacy	Amyloid-related imaging abnormalities with edema among patients receiving bapineuzumab
Donanemab (32)	Anti-Aβ IgG1 targeting established amyloid plaques	Early, prodromal AD	700 mg for the first three doses, followed by 1,400 mg, intravenously, every 4 weeks up to 72 weeks	272	Primary: iADRS. Secondary: CDR-SB, ADAS-Cog, ADCS-iADL, MMSE	Amyloid PET, Tau PET, volumetric MRI	Small reduction of cognitive decline measured by primary outcome in the intervention group, but mixed results in secondary outcomes	ARIA-E occurred in $\frac{1}{4}$ of participants receiving the intervention
Gantenerumab (33)	Anti-Aβ monoclonal antibody that binds with high affinity to aggregated Aβ and promotes its removal by Fc receptor-mediated phagocytosis	Scarlet Road trial: Prodromal	105 mg, 225 mg, or placebo, subcutaneous every 4 weeks	797	Primary: CDR-SB score. Secondary: ADAS-Cog 13, MMSE, Cambridge Neuropsychological Test Automated Battery (CANTAB), FCSRT. Behavioral: NPI-Q, FAQ	Amyloid PET, brain volumes by MRI, CSF Aβ_1−42_, total tau, phosphorylated tau, and neurogranin	No differences between groups in the primary or secondary clinical endpoints were observed	The incidence of generally asymptomatic amyloid-related imaging abnormalities increased in a dose- and APOE ε4 genotype-dependent manner
Gantenerumab (33)	Anti-Aβ monoclonal antibody that binds with high affinity to aggregated Aβ and promotes its removal by Fc receptor-mediated phagocytosis	Scarlet Road trial: Prodromal	105 mg, 225 mg, or placebo, subcutaneous every 4 weeks	797	Primary: CDR-SB score. Secondary: ADAS-Cog 13, MMSE, Cambridge Neuropsychological Test Automated Battery (CANTAB), FCSRT. Behavioral: NPI-Q, FAQ	Amyloid PET, brain volumes by MRI, CSF Aβ_1−42_, total tau, phosphorylated tau, and neurogranin	No differences between groups in the primary or secondary clinical endpoints were observed	The incidence of generally asymptomatic amyloid-related imaging abnormalities increased in a dose- and APOE ε4 genotype-dependent manner
Intravenous immunoglobulin (IVIg) (34)	Intravenous immunoglobulin used in autoimmune disorder and immunodeficiency	Mild to moderate	IV at doses of 0.2 or 0.4 g/kg every 2 weeks or placebo-albumin	390	ADAS-Cog and ADCS-ADL	Volumetric T1-weighted MRI sequences (NeuroQuant). Blood total immunoglobulin; Ab-40 and Ab-42. CSF anti-monomer and anti-oligomer AB assays	No beneficial effects were observed in the dual primary outcome measures	Intervention group had more systemic reactions (chills, rashes) but fewer respiratory infections than participants receiving placebo
Lanabecestat (35)	BACE-1 inhibitor	Mild	20 mg, 50 mg or placebo; oral	AMARANTH trial: 2,218DAYBREAK-ALZ trial: 1,722	ADAS-Cog, ADCS-iADL, FAQ iADRS, CDR-SB, NPI, MMSE	MRI Hippocampal volume, amyloid PET, CSF Aβ_1−42_ and Aβ_1−40_, total tau, phosphorylated tau and soluble Aβ precursor pro- tein (sAPPα and sAPPβ)	Both studies were terminated early after futility analysis	Psychiatric adverse events, weight loss, and hair color changes were reported in a higher percentage of patients receiving lanabecestat
Methylthioninium moiety [leuco-methylthioninium bis (hydromethanesulfonate); LMTM] (36)	Selective inhibitor of tau protein aggregation	Mild to moderate	75 or 125 mg twice daily or 4 mg twice daily (control dose) for blinding urine or fecal discolouration; Oral	891	ADAS-Cog and ADCS-ADL	Volumetric Brain MRI, 18F-FDG-PET. CSF total tau, phospho-tau, and amyloid-β_1−42_	No treatment benefit at either of the doses tested for the coprimary outcomes	Gastrointestinal and urinary effects were the most common adverse events with both high doses of LMTM, and the most common causes for discontinuation
Nilvadipine (37)	Dihydropyridine (DHP) calcium channel blocker	Mild to moderate	8 mg sustained release nilvadipine or matched placebo	511	ADAS-Cog 12, CDR-SB as a gated co-primary outcome		The prespecified primary analyses failed to show any treatment benefit	Higher counts of adverse events on nilvadipine
Verubecestat (38)	BACE-1 inhibitor	Mild to Moderate	12, 40 mg or placebo; oral	1,958	ADAS-cog and ADCS-ADL); MMSE, CDR-SB	MRI hippocampal volume, amyloid PET, CSF total tau	Stopped for futility	
Verubecestat (39)	BACE-1 inhibitor	Prodromal	12, 40 mg or, placebo; oral	1,454	CDR-SB; Progression to the diagnosis of ADdementia	MRI hippocampal volume, amyloid PET, CSF total tau	Stopped for futility	Trend for worse performance in the high dose verubecestat group
Semagecestatv (40)	γ-secretase inhibitor	Mild to moderate	100, 140 mg, or placebo; oral	1,537	ADAS-cog, ADCS-ADL, CDR-SB, NPI, RUD-Lite, EQ-5D, MMSE	CSF Aβ and tau protein, volumetric MRI, amyloid PET	Terminated early. No improvement. Greater cognitive deterioration in the group receiving higher dose	Adverse events: Worsening of cognition, increased rates of skin cancer and infection 76 weeks
Solanezumab (41)	Monoclonal antibody that binds soluble forms of amyloid	Mild to moderate	400 mg every 4 weeks	EXPEDITION 1:1,012EXPEDITION 2:1,040	ADAS-cog; ADCS-ADL	Plasma Aβ_1−40_ and Aβ_1−42_ levels, amyloid PET, CSF total tau and phosphorylated-tau. Volumetric MRI	Neither study showed significant improvement in the primary outcomes	ARIA-E and hemorrhage slightly more frequent with solanezumab
Solanezumab (42)	Monoclonal antibody that binds soluble forms of amyloid	Mild AD	400 mg or placebo; IV every 4 weeks	2,129	ADAS-cog, MMSE, ADCS-ADL, ADCS-iADL, CDR-SB, FAQ, iADRS	Plasma Aβ_1−40_ and Aβ_1−42_ levels, amyloid PET, CSF total tau and phosphorylated-tau. Volumetric MRI	No efficacy	
Tarenflurbil (43)	γ-secretase inhibitor	Mild AD	800 mg or placebo	1,649	Co primary ADAS-cog ADCS-ADL; CDR-SB, Neuro-quality of life scale (QOL-AD), MMSE	Plasma concentrations of S-flurbiprofen	No efficacy	

*PACC: ADCS-ADL-MCI, Alzheimer's Disease Cooperative Study-Activities of Daily Living Inventory; APOE, Apolipoprotein E; ARIA, amyloid-related imaging abnormalities; CDR-SB, Clinical Dementia Rating–Sum of Boxes; CANTAB, Cambridge Neuropsychological Test Automated Battery; DAD, Disability Assessment for Dementia; FAQ, Functional Activities Questionnaire; iADRS, Integrated Alzheimer's Disease Rating Scale; MMSE, Mini-Mental State; NPI-Q, Neuropsychiatric Inventory Questionnaire; PACC, Pre-clinical Alzheimer's Cognitive Composite; RUD-Lite, Resource Utilization in Dementia Lite scale*.

Considering the frustrating results with Aβ targeting therapies, therapeutic development in AD geared to anti-tau drugs, however, with no positive results to date (36). The interest in therapies targeting tau goes beyond AD as other severely debilitating neurodegenerative disorders such as progressive supranuclear palsy (PSP), corticobasal degeneration (CBD), frontotemporal lobar degeneration (FTLD), argyrophilic grain disease, and globular glial tauopathy are also characterized by abnormal deposition of tau protein in the brain (46).

A selection of clinical trials for disease-modification in AD conducted over the past decade is summarized in Table 1. Nearly all recent clinical trials for DMT in AD were led by pharmaceutical industry as part of drug-development initiatives.

In PD, while the accumulation of α-syn is the leading theoretical model, a variety of other homeostatic cell mechanisms have been implicated in the neurodegenerative process and addressed in different clinical trials (Table 2), including autophagy regulation (52–54), mitochondrial dysfunction (55), membrane trafficking (56, 57), calcium homeostasis (58, 59), and neuroinflammation (60). The more specific approaches targeting α-syn are relatively more recent in PD trials than in AD as to date, most studies focused on alternative mechanisms or unspecific protein lowering strategies.

**Table 2:** Recent clinical trials for disease-modification in PD.

**Drug name**	**Mechanism of action**	**Disease stage**	**Dose and administration route**	**Sample size**	**Primary outcome**	**Results**
Exenatide (47)	Antihyperglycemic; glucagon-like peptide 1 (GLP-1) receptor agonist	Moderate PD	2 mg or placebo, subcutaneous injections	62	MDS-UPDRS, motor scores (part 3) in the practically defined off-medication state	Mild improvement in the intervention group in comparison to placebo difference of −3.5 points (−6.7 to −0.3; *p* = 0.0318)
Nilotinib (48)	BCR-ABL inhibitor used for treatment of leukemia	Cohort 1—moderate to advanced PD Cohort 2—early/*de novo* PD	150 or 300 mg; oral	76	Safety and Tolerability; MDS-UPDRS, motor scores part III	Drug was well-tolerated. MDS-UPDRS-part III was worse in the Nilotinib 300 mg group
Pioglitazone (49)	Antihyperglycemic, inhibit the activation of microglia and astrocytes and production of pro-inflammatory cytokines and nitric oxide	Early PD diagnosed within 5 years	15, 45 mg/day, or placebo; oral	210	Total UPDRS score	Negative results
Transdermal Nicotine NIC-PD (NCT01560754)	Agonist of nicotine receptor, modulate dopaminergic function	Early PD up to 18 months of diagnosis	7 or 14 mg nicotine titrated up to 28 mg, or placebo; transdermal	160	UPDRS score (parts 1 to 3) Parkinson's Disease Questionnaire−8 (PDQ-8)	Unpublished
Isradipine (50) STEADY-PD III	Dihydropyridine calcium-channel blocker, used for hypertension	Early PD not requiring symptomatic treatment	5 mg BID or placebo; oral	336	UPDRS score (parts 1 to 3)	Negative results
Inosine (51) SURE-PD3	Urate precursor used for urate elevation	Early PD not requiring symptomatic treatment	Inosine to produce mild elevation of urate; inosine to produce moderate elevation; or placebo	75	Safety and Tolerability; Elevation of urate assessed serially in serum in cerebrospinal fluid	Negative results

*PDQ, Parkinson's Disease Questionnaire; UPDRS, Unified Parkinson Disease Rating Scale*.

The discovery of monogenic forms of PD and genetic risk factors have added more complexity to target identification in PD. For instance, heterozygous mutations in the *glucocerebrosidase* gene (*GBA*), which causes the lysosomal disorder Gaucher's disease when in homozygosis, has been identified as the most common genetic risk factor for PD, and is also associated with more severe cognitive phenotypes in patients with PD (61). These findings emphasized the role of the lysosomal autophagy system in the development and progression of PD, encouraging the investigation of more precise approaches in the selected group of GBA carriers.

Mutations in other genes linked to familial forms of PD, such as the leucine-rick repeat kinase 2 (L*RRK2*), Parkin, and PTEN-induced putative kinase 1 (*PINK1*) brought attention to other disease mechanisms. For instance, while the LRRK2 gene has a role in neurite and synaptic morphogenesis, protein synthesis, membrane trafficking, and autophagy, the Parkin and PINK1 genes participate in mitochondrial health and mitophagy (62). Importantly, neuropathological studies demonstrated that some of these forms of PD did not have the classical disease hallmark, α-syn aggregation (63), raising the question of whether PD is a single disorder or a group of heterogeneous disorders that should be targeted with mechanism tailored interventions (64).

A selection of clinical trials for disease-modification in PD conducted over the past decade is summarized in Table 2. A systematic review previously summarized the studies published before 2009 (65). In general the recent clinical trials for DMT in PD were investigator-initiated and used approved interventions for other conditions, seeking to repurpose it for PD.

## Methodological Challenges in Clinical Trials for Early AD and PD

### Identification of the Study Population

The study population is the subset of the population with the condition of interest defined by the eligibility criteria. In AD and PD trials, the current trend is to recruit patients at the pre-clinical or prodromal stages of these diseases. This approach was encouraged mainly by the recognition that AD and PD pathological processes start decades before the onset of clinical symptoms and the multiple negative trials for DMT in participants with an established diagnosis. However, once individuals in the pre-clinical stages do not have clinical symptoms, the identification of the study population needs to rely on the use of biomarkers and rigorous eligibility criteria. However, biomarkers, either clinical, radiological or laboratorial are not perfect, and one cannot predict with certainty if an individual with positive biomarkers will convert into a clinical phenotype, particularly when there is no gold standard for confirmation of the diagnosis “*in vivo*” (66).

In AD research, biomarkers have been increasingly incorporated over the past decade to confirm that cognitive decline in a given individual is actually associated with AD instead of other differential diagnoses (67). Also, biomarkers have gained crucial importance in identifying individuals in the pre-clinical and prodromal phases of the disease and as surrogate endpoints in the early stages of drug development trials.

In 2018 the National Institute on Aging and Alzheimer's Association (NIA-AA) published a research framework to standardize the use of biomarkers in AD observational and interventional studies (68). The task force emphasized the definition of AD based on the underlying pathological processes instead of clinical presentation, even though the Aβ hypothesis is currently under question, based on the negative results of multiple anti-amyloid RCTs.

The AD biomarkers are labeled according to the nature of the pathologic process that each measure. The biomarkers for Aβ (labeled A) can be based on neuroimaging, amyloid positron emission tomography (PET), or based on CSF measure of Aβ_42_ or Aβ_42_/Aβ_40_ ratio. The biomarkers for aggregated tau/neurofibrillary tangles (labeled T) are the tau PET, or the CSF phosphorylated tau. Furthermore, the group also proposed a biomarker for neurodegeneration or neuronal injury (labeled N), which can be done with imaging using anatomic brain MRI or ^18^F-fluorodeoxyglucose (FDG) PET, or with a measure of total tau in the CSF (68). Based on the abnormal presence of the biomarker (+) or its absence (−) an individual can be classified in different categories, for instance: A- T- N-, means no biomarkers for AD; A+ T- N- means Alzheimer's pathological changes; and A+ T+ with or without N+ means AD (68).

Despite the strong association of the amyloid biomarkers with the pathological process of AD, it is essential to keep in mind that some degree of amyloid accumulation and neurodegeneration are part of the normal aging process, and in many cases, not correlated with the development of cognitive impairment; therefore the prognostic values of these biomarkers are imperfect (69).

In 2019 the Movement Disorder Society (MDS) also updated the proposed research criteria for prodromal PD, which had been previously published in 2015 (70, 71). Because it is not possible to assure with 100% certainty that someone with prodromal PD will convert into a clinical phenotype, the MDS proposed the use of likelihood ratios, positive (LR+) and negative (LR–), based on all available evidence for a given biomarker. The prodromal markers with the highest LR+ include polysomnography confirmed Rapid-eye-movement sleep behavior disorder (RBD), abnormal dopaminergic PET/SPECT, orthostatic hypotension, presence of subthreshold parkinsonism, and olfactory loss. For the carriers of genetic biomarkers in the *GBA* and *LRRK2* genes, the LR+ is dependent on age, i.e., higher in individuals after 70 years-old (71).

Several other biomarkers appear to be potentially useful to identify individuals at the prodromal stage of PD, including the identification of phosphorylated α-syn in the skin and submandibular glands. However, the sensitivity and specificity of these markers can be variable according to the methodology used (71). Also, neuroimaging biomarkers have promising value in assessing the PD Braak stages 1 to 3, including the ^11^C-donepezil PET and CT showing the reduced uptake of ^11^C-donepezil in the colon, the ^123^I-metaiodobenzylguanidine (MIBG) scintigraphy of the heart showing sympathetic denervation, the susceptibility-weighted and neuromelanin-sensitive MRI of locus coeruleus and substantia nigra, and the ^11^C-methylreboxetine (MeNER) PET showing decreased thalamic MeNER binding potential (72). These methods, however, have not been fully validated for inclusion in the criteria for prodromal PD (71).

Despite these various instruments to identify the study population with prodromal AD and PD, these are not gold-standard diagnostic methods. Besides, these biomarkers need to be used in combination, therefore forming prediction models (73).

A prediction model is a formal combination of individual predictors from which risks of specific outcomes can be calculated for a given individual. In the case of AD and PD, a useful model should provide accurate predictions to inform investigators of the chance of an individual converting into clinical forms of the disease (73). This is a real challenge, because a different combination of biomarkers to compose the prediction models have been proposed, which were validated and tested in specific study populations, sometimes with no external and independent validation, and often using small samples. Thus, carrying several sources of bias (74). Taking that into account, there is a high chance that studies in pre-clinical and prodromal AD and PD may enroll a very heterogeneous group of participants, making the detection of a true signal harder.

### Definition of Primary Outcomes

The choice of primary outcomes is a crucial point in RCT design. The outcome basically reflects the primary question of the study, which postulates that in a given study population, an intervention, in comparison to a control, will lead to a given outcome (75). Ideally, the outcome needs to be relevant to patients, passive of unbiased assessment, and potentially influenced by the intervention (76). An essential challenge in the design of DMT trials in prodromal or pre-clinical stages of AD and PD is that, by definition, the subjects will not have established symptoms at baseline. Therefore, several practical challenges arise: How to measure disease progression with no apparent symptoms? What is considered relevant to patients in those circumstances? How to differentiate the effects of the intervention from disease characteristics and compensatory mechanisms?

Although a large number of biomarkers has been suggested to identify pre-clinical and prodromal phases of AD and PD, in general, the utility of these biomarkers as surrogate primary outcome measures is uncertain (77). Traditionally, rating scales that measure the severity of symptoms have been selected as primary outcomes, even in cases of early disease stages (Tables 1, 2).

In AD trials, most studies have used the Alzheimer's Disease Assessment Scale—Cognitive (ADAS-Cog), which was designed in 1984 and has been successfully used in clinical trials for symptomatic therapies, such as cholinesterase inhibitors. The ADAS-Cog assesses deficiencies in episodic memory, language, orientation, praxis, and other domains affected in AD. Despite its use in many DMT trials over the past decade (Table 1), there is a concern that this instrument may not be sensitive to mild cognitive changes; therefore, it would not be ideal for pre-clinical and prodromal stages of AD (78). To improve the accuracy of the ADAS-Cog in early disease phases, supplementary items have been added to the scale, which is now available in 11-, 12-, 13-, and 14-item versions (78, 79). Recent trials have selected these versions as primary outcomes, however, to date, no DMT RCTs have found statistical significant differences in the ADAS-Cog between the intervention and control groups (Table 1).

Psychometric evaluations of the ADAS-Cog suggest that despite the adequate reliability, validity, and scaling assumptions, in patients with MCI, the detection of changes is limited by a substantial floor effect (80). For instance, patients with MCI have an average score of 11 to 12 (standard deviation of 4) out of a possible range of 0 to 70. Also, the minimal clinically important difference (MCID) using physician judgment as an anchor was estimated to be of 3 points-decline in a sample with established AD (81), which may be substantial for patients in pre-clinical and prodromal stages.

Another scale frequently used in AD clinical trials is the Clinical Dementia Rating scale (CDR) sum of boxes (CDR-SB), which assesses cognitive dysfunction and functional ability. The CDR-SB has demonstrated to correctly classify patients across a spectrum of MCI to dementia, with a good agreement, κ of 0.91 (82). However, it also appears susceptible to floor and ceiling effects to differentiate healthy controls from MCI and prodromal AD, thus lacking sensitivity to detect changes in very early, i.e., pre-clinical and prodromal stages of the disease, unless the follow-up is long and the sample size is large (78). In line with that, the MCID for the CDR-SB was estimated between 1 to 2 point-increase out of a maximum range of 0–18 (83), which is a considerable change to be observed in the early stages of AD.

In 2018 the Food and Drug Administration (FDA) released a guidance for the industry to develop drugs for treatment of early AD. The statement suggested the classification of AD according to 4 stages. For each of these stages, the FDA suggested endpoints to be used in clinical trials, however, without recommending specific measures or rating scales. The proposed stage classification and the characteristics of the recommended outcomes required for approval consideration are summarized in Table 3 (84).

**Table 3:** FDA suggestions for endpoints in clinical trials for early AD.

**Clinical trial population—AD stage**	**Definition according to clinical and biomarker characteristics**	**Characteristics of recommended endpoints**
Stage 1	Pathophysiological features demonstrated by assessment of various biomarkers measures in completely asymptomatic participants	•An effect on the characteristic pathophysiological changes of AD, as demonstrated by an effect on various biomarkers •Alternative approach would include to perform a study long enough to evaluate measures recommended for stage 2
Stage 2	Characteristic pathophysiological features of AD on biomarkers, and subtle detectable abnormalities on sensitive neuropsychological assessment without functional impairment	•Consider studies with significant duration to allow endpoints suggested for stage 3 •Sensitive measures of neuropsychological performance, requiring consistency across multiple individual tests, or a large magnitude effect •Additional arguments for approval be predicated on certainty of diagnosis, certainty of expected clinical course, and certainty of the relationship of the observed effects and characteristic pathophysiologic changes
Stage 3	Characteristic pathophysiological features of AD on biomarkers, and subtle or more apparent abnormalities on sensitive neuropsychological measures, and mild functional impairment, which is but detectable but not severe enough for dementia diagnosis	•Outcome will provide an assessment of meaningful cognitive function •Integrated scale that adequately and meaningfully assesses both daily function and cognitive effects •Independent assessment of daily function and cognitive deficits is an acceptable approach
Stage 4	Patients with overt dementia	•Discussion on outcomes at these stages were not included on the FDA statement

The Unified Parkinson Disease Rating Scale (UPDRS) or the most recent version, MDS-UPDRS, has been the outcome of choice in PD trials (85). The scale is composed of parts 1 to 4, which, respectively, measure the mental status, the activities of daily living, the motor examination scores, and the complications of therapy.

The motor scores of the UPDRS (i.e., part 3) has been successfully used as the primary outcome in trials for symptomatic therapies over decades. The scale ranges from 0 to 108, the higher, the more severe parkinsonism, and the MCID has been estimated between 5 to 6 point-decline with symptomatic treatment (86). However, it is unclear if this outcome is appropriate to detect disease modification. One important caveat is that the motor UPDRS significantly changes with the introduction and adjustment of symptomatic therapy; therefore, changes in therapy during the trial might affect the score (9). To minimize this issue, investigators opt to assess the outcome in the practically defined “off-medication” state, i.e., after 12-h medication withdrawal. Still, it is possible that a long-acting carryover effect might influence the scores, although unlikely (87).

Alternatively to motor scores, some DMT trials in PD have used the sum of scores from parts 1 to 3, aiming to encompass mental status and functionality (Table 2). In a limited number of DMT studies, a measure of PD related quality of life such as the Parkinson's Disease Questionnaire (PDQ-39) has been included; however, this scale was found to be biased toward more severe problems, posing limitations to its use in RCTs aiming to detect small but clinically relevant changes in patients with early phases of the disease (88).

Neuroimaging-based biomarkers, such as dopamine transporter scans (DaT-Scan) and 18_F-DOPA PET have been increasingly proposed as surrogate exploratory outcomes in PD clinical trials. Indeed, neuroimaging tools such including 18_F-DOPA PET, FDG PET and Fluoro-m-tyrosine PET have been successfully used in gene therapy studies in PD (89). However, the inconsistent correlation between imaging findings and clinical changes, have limited a broader implementation of these technologies as relevant outcomes in PD trials (89).

Overall, the choice of outcomes in RCTs for DMT in PD and AD have largely neglected patients' perspectives. This is not a trivial problem, particularly in an era when patients and other stakeholders are increasingly involved in medical research, and in this population enriched with older individuals who tend to have comorbidities and complex health and social issues (90). Moreover, the outcomes measures used in DMT trials appear to have been “imported” from symptomatic trials, which represents an important source of problems. Pre-clinical and prodromal participants have much less severe symptoms than participants in symptomatic trials. Also, the timeline for potential disease-modifying effects of an intervention is much longer than the observed for symptomatic effects, thus requiring a prolonged follow-up and very large samples for the detection of small changes.

### Sample Size, Statistical Analysis, and Duration of Follow Up

Even the most well-designed research question will be left unanswered if the study is underpowered. Undersized trials may end with negative results that are not interpretable, while oversized studies may find statistically significant differences that are not clinically relevant. To adequately estimate the sample size, several characteristics of the study need to be taken into account, including the experimental design, the study hypothesis, the nature of the outcome, the statistical test, the MCID, the significance threshold and power, and the expected attrition (91).

In AD, most trials have a parallel group design comparing intervention to placebo (2 groups) or two different doses of the intervention and placebo (3 groups). The trials aimed to demonstrate the superiority of the intervention against placebo, typically using one of the rating scales previously discussed as continuous outcome measures. Because of the small changes expected in the outcome scales in participants at pre-clinical and prodromal stages, relatively large sample sizes have been required in AD trials, ranging from 1500 to 2000 (Table 1).

The recruitment and retention of such large samples represent an important challenge for several reasons: Individuals at prodromal and particularly at pre-clinical stages typically will not be aware of their potential candidacy for clinical trials; the identification of the eligible population require screening of a considerable number of participants with costly and invasive biomarkers, leading to a high-screen rate failure; and the studies require long follow-ups which are associated with attrition in this population due to withdrawal of consent and comorbidities (77). The AD studies have been planned to detect changes after an average 78 weeks of treatment, however, many studies were discontinued early either because of futility or adverse events. While the futility analysis may reduce costs and trial duration with an intervention that is likely ineffective, it carries the inherent risk of pre-mature early discontinuation for modest effects (77).

In PD, a similar pattern of parallel design trials has been conducted to detect differences between potential DMT and placebo, however with smaller samples. Classical studies have used the “delayed start design” to evaluate disease-modification with early vs. delayed introduction of symptomatic interventions (65), and a single pioneer study used the factorial design (92). PD has a slow disease progression, and the studies have been planned for an average 2-year follow-up, which may be enough to detect changes in the UPDRS in patients in the clinic phase of the disease, but may be insufficient to detect changes in the prodromal stages on the disease.

## Perspectives for Clinical Trials for Early AD and PD

Designing an RCT is definitely not an easy task. This is particularly more challenging when dealing with pre-clinical and prodromal phases of neurodegenerative disorders. By looking at the recent research in the fields of AD and PD, clinical trials in AD appeared to have achieved more sophistication, largely due to the advances in biomarker research. For that reason, AD research is closer to move to studies in the pre-clinical stage of the disease. On the other hand, the PD field is only starting to look at the prodromal stages of the disease and more questions needs to be addressed before moving research to the pre-clinical stages. Independent of these differences in timing, similar issues are faced in both areas.

### Innovations in Study Design

Beyond the traditional parallel group design, alternative designs can be more efficient in terms of cost and duration. One of these designs for instance, is the 2 × 2 factorial design, in which two interventions can be simultaneously tested. However, in the case of drug development trials, in which response to each intervention and adverse events need to be strictly monitored, factorial design appears to be inappropriate. Contrarily, for RCT testing approved drugs for repurposing or repositioning for disease-modification or other types of therapies, such as diet modification or physical exercise, when the interaction between the interventions is unlikely to result in severe adverse events, this type of design can be very useful (91).

The option of adaptive design has been increasingly used in other research fields, for instance in oncology (93). An adaptive trial allows for pre-specified modifications during the trial based on interim data analysis. Some potential adaptations include sample size reassessment, responsive adaptive allocation, seamless designs and enrichment designs (94). The seamless design may be particularly useful for drug development in AD and PD, as it allows for transition from a phase 2 to a phase 3 RCT. Similarly, the enrichment design could be beneficial in AD and PD trials, because it allows for changes in eligibility criteria when a subgroup with a certain characteristic or biomarker shows more promising results (94). This approach would have a great potential to mitigate the population heterogeneity problem in AD and PD studies, allowing for enrichment based on genotype or other biomarkers. It is important to note that these adaptation can be parsimoniously combined in a single trial, thus simultaneously addressing multiple issues encountered in AD and PD RCTs (95).

Another alternative is the master protocol design, also called platform trials. These studies have a single overarching protocol developed to evaluate multiple hypotheses, with the general goal of improving efficiency (96). The platform basket trials, which evaluate a single targeted therapy on multiple diseases; or umbrella trials, which test multiple interventions in a single disease (96) can be feasible option to test a single drug in multiple neurodegenerative processes, for instance AD and PD, or to test one drug in multiple disease, for instance, an anti-tau therapy and AD and other tauopathies.

### Improving Eligibility Criteria

The solutions for dealing with the heterogeneity of the study populations in AD and PD studies, particularly in the pre-clinical and prodromal stages, invariably depend on the development and validation of more precise prediction models (73), and assessment of quality of evidence using appropriate approaches, such as the Grades of Recommendation, Assessment, Development, and Evaluation (GRADE) system (97).

An important debate is whether a more pragmatic or mechanistic approach should be used in disease-modifying trials. Pragmatic trials would include large number of participants independent of underlying disease mechanisms, genotypes, biomarker profiles (98). However, at the expense of enrolling participants with different subtypes of disease, some of each unresponsive to the mechanism of the intervention. These trials are useful to inform real-world decisions, which appears to be a step further in RCT for DMT in AD and PD.

Considering that AD and PD have multiple pathophysiological mechanisms, and that the exact role of each of these mechanisms in disease progression is somewhat unclear, trials focused on the mechanistic approach would be more beneficial at this initial stage. For instance, studies focusing on participants with primary progressive aphasia or in carriers of specific genetic variants, would allow for more homogenous samples, likely sharing similar disease mechanisms. However, the generalizability of the results could be limited, and further studies would be necessary before extrapolating the findings to a more heterogenous population with AD or PD. Once again, the success of this approach would be dependent of development of biomarkers for the disease mechanism under target (9), which would ultimately allow enriching and stratification of the study population.

### Designing Primary Outcomes

The lack of a single comprehensive instrument that meets the criteria for an optimal primary outcome in disease-modification trials is a main limiting factor in RCT designs for early AD and PD.

In trials enrolling pre-clinical or prodromal participants, the most simplistic approach would be to use survival analysis or Cox regression models to measure time-to-event, i.e., time to development of symptoms (phenoconversion) or to fulfill clinical diagnostic criteria, respectively. This approach has been successfully used across several disciplines. However, it may be challenging to implement in trials for AD and PD due to the slow progression of these conditions, therefore, requiring a long follow up to detect between group differences (32).

Another potential approach would involve the design of a good composite outcome, for instance, using a clinical scale, a measure of patient preference, and a clinically relevant biomarker. The use of composite outcomes may substantially increase the efficiency of clinical trials, however important questions need to be considered when combining outcomes: Do the outcome components: Have similar importance? Respond similarly to the intervention? Occur in the same frequency? (99, 100). If these requirements are not met, it is better not to combine these outcomes, but use each separately or as co-primary outcomes.

### Digital Innovations in Clinical Trials

Recently, the use of technology-based objective measures has gained substantial attention in neurological disease research. These innovative digital monitoring tools include wearable, portable, body-fixed sensors, or domestic-integrated devices that collect continuous or frequent, objective, and multidimensional data during daily activities, providing a more ecologic and reliable measure of patients' cognition, functionality, and mobility (101). These digital biomarkers can potentially be useful in several stages of clinical research.

In the recruitment phase, the use of digital sensors may allow for the detection of subtle symptoms in the early stages of the disease, i.e., improving the detection of subjects at the prodromal phases of AD and PD, before symptoms can be clinically documented. In addition, in conjunction with big-data analysis and artificial intelligence tools, these objective measures may be used to predict the likelihood of an individual to convert into a clinical phenotype within a certain time frame, therefore allowing for the screening and inclusion of patients more likely to experience the outcome of interest, which could reduce trial duration and costs (101).

The mobile health technology may also have a role in facilitating patient engagement and compliance, by allowing the continuous monitoring of symptoms and disease progression in participants' natural environment, during patient-relevant daily activities, instead of the artificial observation in the supervised environment, like in the clinic. Similarly, these digital measures can be used in exploratory outcomes to support primary endpoints measured in more traditional ways. Indeed, this approach has been encouraged by regulatory agencies, such as the FDA (102). At this point, there is not enough information and evidence to support the consideration of digital biomarkers as primary outcomes in clinical trials (48, 102, 103).

Despite the great potential for the use of digital biomarkers, several issues have limited the adoption of these technologies into clinical practice and clinical trials, including: the weak correlation found between digital biomarkers and results from clinical assessments, the difficult validation of algorithms used by the devices, and the approaches required to analyze the massive amount of data generated by the continuous or frequent monitoring of multiple simultaneous data (103).

## Conclusions

There is a crucial need for effective DMT in AD and PD. Considering the potential dissemination of the pathological processes of these conditions by the time of the clinical diagnosis, future clinical trials need to be designed for patients at much earlier stages of these diseases, i.e., in the pre-clinical or prodromal stages. The rapid advances in disease biomarkers now allow the identification of individuals at risk for these disorders before clinical diagnostic criteria are fulfilled, thus allowing for early recognition of potential candidates for clinical trials. Despite that, designing and implementing trials focused on early disease stages has several challenges.

One of such challenges is reliably identifying the ideal study population based on the currently available biomarkers. More importantly, how to accurately predict the risk of phenoconverion to minimize heterogeneity and enrich the clinical trial population? When it comes to defining primary outcomes, another challenge is faced: monitoring disease progression in subjects with no clinically established manifestations of the disease. The clinical rating scales applied in several trials appear to be insensitive to subtle changes in early disease stages; thus, innovative approaches including neuroimaging and digital biomarkers combined with sophisticated and patient-relevant disease measures appear to be more promising strategies. Finally, the need to optimize trial efficiency and cost is another relevant issue. This may be at least partially improved by employing adaptive trial designs, bioinformatics tools, and validated predictive models of disease in clinical trials.

Obviously, not even the most sophisticated methodological innovations will overcome the currently incomplete understanding of AD and PD pathophysiological mechanisms. Therefore, it remains essential to refine the knowledge on these diseases, to improve the selection of more promising interventions for future clinical trials.

## Author Contributions

CA was responsible for the conceptualization, design, draft, and review of the manuscript.

## Conflict of Interest

The author declares that the research was conducted in the absence of any commercial or financial relationships that could be construed as a potential conflict of interest.

## Publisher's Note

All claims expressed in this article are solely those of the authors and do not necessarily represent those of their affiliated organizations, or those of the publisher, the editors and the reviewers. Any product that may be evaluated in this article, or claim that may be made by its manufacturer, is not guaranteed or endorsed by the publisher.
